# Fifty years of immunization in Africa: reflecting on the past, celebrating progress, and shaping the future

**DOI:** 10.11604/pamj.supp.2025.51.1.48177

**Published:** 2025-06-11

**Authors:** Charles Shey Wiysonge

**Affiliations:** 1 Vaccine-Preventable Diseases Programme, World Health Organization Regional Office for Africa, Brazzaville, Congo

**Keywords:** Immunization, Addis Declaration on Immunization, Immunization Agenda 2030, Africa

## Editorial

Immunization is universally acknowledged as one of the most impactful and cost-effective public health interventions in human history [1, 2]. It averts illness and death on a vast scale. It leads to an extraordinary return on investment of up to 52 United States (US) dollars for every dollar spent on childhood immunization [3]. The World Health Organization (WHO) published the first immunization schedule in 1961, calling for vaccination of both infants and adolescents, following discussions on the importance of immunization at the 13th World Health Assembly in 1960 [4]. More than a decade later, in 1974, the concentrated global effort to use immunization as a public health intervention began; when WHO launched the Expanded Programme on Immunization (EPI) and recommended vaccination against seven diseases, including diphtheria, measles, pertussis, poliomyelitis, smallpox, tetanus, and tuberculosis [5].

We still vaccinate against six of the original seven EPI diseases today, following cessation of vaccination against smallpox after the eradication of this dreadful disease that used to maim and kill millions of people each year. There has been substantial progress in expanding national immunization schedules in Africa, especially following the creation of Gavi, the Vaccine Alliance in 2000, with most countries now routinely vaccinating against a dozen or more diseases [6]. The most recent demonstration of Africa’s ability to rapidly introduce and roll out new vaccines is the accelerated introduction and rollout of malaria vaccines since January 2024 [7]. From 2019 to December 2023, only three countries (Ghana, Kenya, and Malawi) that participated in the pilot Malaria Vaccine Implementation Programme were using malaria vaccines in Africa. Between January 2024 and May 2025, a total of 17 countries on the continent introduced malaria vaccines, a massive feat.

In Africa, the EPI has been a cornerstone of disease prevention, catalyzing broader improvements in child survival, health systems, and health equity [1,8]. From 1974 to 2024, vaccination efforts in Africa prevented more than 50 million deaths and averted more than three billion disability-adjusted life years (DALYs), demonstrating the transformative impact of immunization on population health [9]. DALYs quantify the total healthy life years lost due to premature death and years lived with disability [10]. A DALY refers to the loss of the equivalent of one year of full health. The impact of immunization has been so dramatic in the last five decades that it is now easy to forget that diseases like smallpox, polio, yellow fever, and others, used to cause millions of deaths and disabilities each year in many parts of the world (including in Africa) that are now free of these diseases, thanks to immunization [11].

Africa´s immunization landscape has evolved remarkably, yet major inequities persist. The COVID-19 pandemic severely disrupted routine immunization services, exposed deep structural vulnerabilities, and fuelled vaccine hesitancy. Each year since 2020, more than nine million children less than one year of age (equivalent to more than one-quarter of surviving infants) in Africa do not get immunized, including zero-dose children who have not yet started their vaccination series and under-vaccinated children who started but did complete all the vaccination doses in the schedule. These immunity gaps vary widely between and within countries, leading to frequent outbreaks of vaccine-preventable diseases (VPD), including measles, circulating vaccine-derived polioviruses, diphtheria, yellow fever, and others. Despite substantial investments in supplementary immunization activities (SIAs), persistent measles outbreaks highlight critical weaknesses in both second-dose measles coverage and SIA implementation [8]. With national health systems experiencing increasing pressures from economic shocks and conflict to climate change and vaccine misinformation, the need to invest in resilient and equitable immunization systems cannot be over-emphasized [1,11].

This special journal supplement offers strategic and scientific reflections on the evolution of immunization on the African continent. The aim is to take stock, document, and share country experiences, and chart a methodical way forward. The journey ahead must be anchored in the Addis Declaration on Immunization (ADI) and the Immunization Agenda 2030 (IA2030). The ADI, endorsed by African Heads of State in January 2017, is a historic pledge to prioritize immunization through stronger political commitment, domestic investment, and accountability [12]. The Framework for the Implementation of the IA2030 in the WHO Africa Region, adopted by African Ministers of Health in August 2021, envisions an Africa where everyone, everywhere, at every age benefits fully from vaccines for health and well-being. The framework sets clear targets to reduce VPDs, promote equity, and strengthen integration with primary health care in Africa by 2030 [8].

Six strategic imperatives stand out, including, (1) reaching zero-dose and under-vaccinated children; (2) expanding access to new and under-utilized vaccines through coordinated regional rollouts; (3) advancing measles and rubella elimination; (4) leveraging implementation research to strengthen immunization decision-making; (5) strengthening programme foundations, including robust surveillance, supply chains, health workforce capacity, and real-time data systems; and (6) reinforcing political leadership and accountability. The Big Catch-Up initiative, launched in 2023, provides a critical framework for countries to recover lost ground and strengthen systems to prevent future disruptions [8]. A renewed regional verification mechanism, coupled with outbreak analytics and tailored responses, is needed to make measles and rubella elimination a reality in Africa. In addition, we should leverage implementation science on the continent to guide a fit-for-purpose learning agenda and facilitate prompt knowledge translation of useful strategies across contexts in a manner that allows replication with fidelity [13]. Furthermore, political will in Africa must translate into sustained budg *et al* locations and policy coherence at national and subnational levels [12].

As immunization evolves into a life-course intervention from infancy through adulthood in Africa, it must remain firmly embedded in universal health coverage and primary health care. This special journal supplement is not just a retrospective, it is a rallying cry for the future. The supplement brings together the evidence, the voices, and the insights needed to write the next chapter of immunization in Africa. Immunization has saved more lives in Africa than any other health intervention. As we honour five decades of extraordinary impact, we should not be content with celebration alone. The path ahead requires bold commitments, equity-driven strategies, and collective resolve. The targets for 2030 are within reach - but only if we act with urgency, with clarity, and with sustained solidarity. We should do this with a collective vision that goes beyond 2030. Our gaze - as we press forward - must lift beyond the horizon of 2030, to a future where the promise of immunization touches every life, in every place, and for every generation to come in Africa.
